# Abstracts from the 11th Symposium on Experimental Rhinology and Immunology of the Nose (SERIN 2017)

**DOI:** 10.1186/s13601-017-0163-x

**Published:** 2017-08-23

**Authors:** Ibon Eguiluz Gracia, Carmen Rondón, Paloma Campo, Ana Prieto, Lina Mayorga, Luisa Galindo, Ana Molina, Miguel Blanca, Maria Jose Torres, Taiyo Morikawa, Ayumi Fukuoka, Kazufumi Matsushita, Shigeharu Fujieda, Tomohiro Yoshimoto, Naruhito Iwasaki, Tamar Smulders, Danielle Van Egmond, Kees Van Drunen, Marc Van Der Schee, Achim Georg Beule, Margot Berings, Anton Jult, Hanne Vermeulen, Natalie De Ruyck, Lara Derycke, Hakan Ucar, Philip Ghekiere, Robin Temmerman, John Ellis, Claus Bachert, Bart Lambrecht, Melissa Dullaers, Philippe Gevaert, Stefania Arasi, Serena Perna, Yvonne Resch, Christian Lupinek, Kuan-Wei Chen, Susanne Vrtala, Rudolf Valenta, Paolo Maria Matricardi, Ivânia Gonçalves, Tiago Jacinto, Rita Amaral, Ana M. Pereira, Luís M. Araújo, Mariana Couto, João A. Fonseca, Par Stjarne, Ranbir Kaulsay, Wolfgang Pohl, Maria Carmen Plaza, Ana Maria Prieto, Cristobalina Mayorga, Magdalena Herknerova, Tengchin Wang, Chiejun Wu, Jonathan Kilimajer, Laura Pujols, Jordi Roca-Ferrer, Borja Callejas, Mireya Fuentes-Prado, Maria Perez-Gonzalez, Isam Alobid, Antonio Valero, Cesar Picado, Ruth Murray, Joaquim Mullol, Brecht Steelant, Katleen Martens, Guy Boeckxstaens, Sven F. Seys, Peter W. Hellings, Timothy C. Biggs, Stephen M. Hayes, Philip G. Harries, Sylvia Pender, Rami J. Salib, Jean Kim, Hyun Sil Lee, Livije Kalogjera, Nada Vrkic, Anita Topic, Dejan Tomljenovic, Tomislav Greguric, Patricija Bankovic Radovanovic, Rainer Jund, Pascal Haimerl, Adam M. Chaker, Yvonne Schober, Sonja Schindela, Andreas Nockher, Carsten B. Schmidt-Weber, Julia Esser-Von Bieren, Pascal Ickrath, Norbert Kleinsasser, Niklas Beyersdorf, Xin Ding, Rudolf Hagen, Stephan Hackenberg, Daniela Cangiano, Francesco Cinetto, Giuseppe Brescia, Gino Marioni, Claudia Zanotti, Franco Schiavon, Roberto Padoan, Ilaria Caputo, Raffaella Neri, Carlo Agostini, Ji Heui Kim, Yong Ju Jang, Ji Youn Lim, Sung Hee Kim, Elena Savlevich, Leonid Gaganov, Maria Kochnova, Victor Egorov, Jie Shen Fok, Tanzeela Hanif, Jutta Renkonen, Sakari Joenväärä, Matti Kankainen, Mika Mäkelä, Paula Kauppi, Anna Pelkonen, Pirkko Mattila, Risto Renkonen, Sanna Toppila-Salmi, Gabriele Holtappels, Bart N. Lambrecht, Natalia Blanca-López, Miguel Gonzalez-Visiedo, Raquel Jurado, Gabriela Canto

**Affiliations:** 1 Hospital Regional Universitario de Malaga and IBIMA, Málaga, Spain; 2 Hyogo College of Medicine, Nishinomiya, Hyogo Japan; 3 University of Fukui, Fukui, Japan; 4 AMC-UvA, Amsterdam, The Netherlands; 5 Klinik für Hals, Nasen- und Ohrenheilkunde, Universitätsklinikum Münster, Münster, Germany; 6 Upper Airways Research Laboratory, Ghent University, Ghent, Belgium; 7 Ghent University, Ghent, Belgium; 8 BekaertDeslee Innovation bvba, Waregem, Belgium; 9 Chrisal NV, Lommel, Belgium; 10 Devan Chemicals NV, Ronse, Belgium; 11 Laboratory of Immunoregulation, VIB Inflammation Research Center, Ghent University, Ghent, Belgium; 12 Department of Pediatric Pneumology and Immunology, Charité Medical School, Berlin, Germany; 13 Medical University of Vienna, Vienna, Austria; 14 CUF Porto - Instituto & Hospital, Porto, Portugal; 15 CUF Porto - Instituto & Hospital, CINTESIS - Center for Health Technology and Services Research, ESS - IPP - School of Health, Polytechnic of Porto, Porto, Portugal; 16 CUF Porto - Instituto & Hospital, CINTESIS - Center for Health Technology and Services Research, Porto, Portugal; 17 CUF Porto - Instituto & Hospital; Allergy Unit, FMUP - Faculty of Medicine, University of Porto, Porto, Portugal; 18 CUF Porto - Instituto & Hospital, CINTESIS - Center for Health Technology and Services Research, MEDCIDES - Department of Health Information and Decision Sciences, Faculty of Medicine, University of Porto, Porto, Portugal; 19 Karolinska Institute, Stockholm, Sweden; 20 Clontarf Clinic, Dublin, Ireland; 21 Karl Landsteiner Institut fur Experimentelle und Klinische Pneumologie, Vienna, Austria; 22 Allergy Unit, Malaga, Spain; 23 IBIMA, Malaga, Spain; 24 Research Laboratory, Malaga, Spain; 25 Regional University Hospital of Malaga, Malaga, Spain; 26 UMA, Malaga, Spain; 27 Hospital Na Homolce, Prague, Czech Republic; 28 Department of Otolaryngology, Tainan Municipal Hospital, Tainan City, Taiwan; 29 Department of Pathology, Tainan Municipal Hospital, Tainan City, Taiwan; 30 Subiza Asthma and Allergy Center, Madrid, Spain; 31 IDIBAPS & CIBERES, Barcelona, Spain; 32 IDIBAPS, CIBERES & University of Barcelona Hospital Clinic, Barcelona, Spain; 33 Medscript, Dundalk, Ireland; 34 KU Leuven, Louvain, Belgium; 35 University of Southampton NHS Foundation Trust, Southampton, UK; 36 Johns Hopkins University School of Medicine, Baltimore, MD USA; 37 ORL/HNS Department, University Hospital Centre, Zagreb, Croatia; 38 Biochemistry Laboratory, University Hospital Centre, Zagreb, Croatia; 39 Clinical Institute of Chemistry, Zagreb, Croatia; 40 Clinical Department of Radiology, University Hospital Centre, Zagreb, Croatia; 41 Department of Clinical Chemistry, Genera Hospital Pula, Pula, Croatia; 42 URL, Ghent, Botswana; 43 Center of Allergy and Environment (ZAUM), Technical University of Munich and Helmholtz Center Munich, Munich, Germany; 44 Department of Otolaryngology, Allergy Section, Klinikum Rechts der Isar, Center of Allergy and Environment (ZAUM), Technical University of Munich, Munich, Germany; 45 Institute of Laboratory Medicine and Pathobiochemistry, Molecular Diagnostics, Philipps University Marburg, Marburg, Germany; 46 Department of Otorhinorhinology, University of Wuerzburg, Würzburg, Germany; 47 Institute of Immunology and Virology, Würzburg, Germany; 48 Department of Medicine DIMED, University of Padua, Padua, Italy; 49 Department of Neurosciences DNS, Otolaryngology Section, University of Padua, Padua, Italy; 50 Department of Internal Medicine, University of Padua, Padua, Italy; 51 University of Padua, Padua, Italy; 52 Asan Medical Center, Seoul, South Korea; 53 Central State Medical Academy of Department for Presidential Affairs of the Russian Federation, Moscow, Russia; 54 Moscow Regional Research and Clinical Institute (MONIKI), Moscow, Russia; 55 Flinders Medical Centre, Bedford Park, Australia; 56 University of Helsinki, Helsinki, Finland; 57 Allergy Unit, Regional University Hospital of Malaga UMA, Málaga, Spain; 58 Allergy Service, Hospital Infanta Leonor, Madrid, Spain; 59 Research Laboratory, IBIMA, Regional University Hospital of Malaga UMA, Málaga, Spain

## Oral Abstract Session 1: Rhinitis

### O01 Long-term follow-up of local allergic rhinitis patients

#### Ibon Eguiluz Gracia, Carmen Rondón, Paloma Campo, Ana Prieto, Lina Mayorga, Luisa Galindo, Ana Molina, Miguel Blanca, Maria Jose Torres

##### Hospital Regional Universitario de Malaga and IBIMA, Málaga, Spain

**Correspondence**: Ibon Eguiluz Gracia - iboneguiluz@gmail.com

*Clinical and Translational Allergy* 2017, **7(Suppl 3)**:O01

**Introduction**: There are few data available regarding natural history of local allergic rhinitis (LAR). We previously reported the results of the first 5-years of follow-up observing a similar rate of development of systemic allergic rhinitis (AR) in both LAR patients and healthy controls.

**Objective**: To explore the natural history of a population with LAR and the development of AR and comorbidities over a 10 year period.

**Methods**: A cohort of 194 patients with LAR of recent onset (<18 months) and 130 age- and sex-matched healthy controls were prospectively evaluated in a 10-year follow-up study (2005–2016). All participants provided informed consent and ethic committee of the hospital approved the study. Clinical-demographic questionnaire, spirometry, SPT and specific IgE (sIgE) to aeroallergens were evaluated yearly. Nasal allergen provocation tests (NAPT) with D. pteronyssinus (DP), Alternaria, Olea europea, and grass pollen were performed at baseline, and after 5 and 10 years.

**Results**: A total of 151 patients (78%) and 90 controls (69%) completed the study. At baseline, most patients had moderate-to-severe persistent-perennial rhinitis. Conjunctivitis (52%) and asthma (19%) were the main comorbidities, and DP the most frequent sensitizing aeroallergen (51.1%). During the 10 years of evaluation 21 new cases of asthma (12.5%, P = 0.007) and 17 new cases of conjunctivitis (10%, P = 0.067) were diagnosed. After 10 years of evolution a similar rate of development of AR was detected in patients and healthy controls (11.3 vs 10%, P = 0.761). In 5 patients, conversion to systemic atopy occurred in the last year of evaluation (3%).

**Conclusions**: LAR is a well-differentiated clinical entity with a low rate of development of systemic atopy. This study was funded by the Institute of Health “Carlos III” of the Spanish Ministry of Economy and Competitiveness through the RETICS ARADyAL (RD16/0006/0001) and the FIS PI14/00864.

**Keywords**: Local allergic rhinitis, Allergic rhinitis, Sensitization

### O02 ILC2-activation aggravates Th2-dependent nasal inflammation in mice

#### Taiyo Morikawa^1^, Ayumi Fukuoka^1^, Kazufumi Matsushita^1^, Shigeharu Fujieda^2^, Tomohiro Yoshimoto^1^

##### ^1^Hyogo College of Medicine, Nishinomiya, Hyogo, Japan; ^2^University of Fukui, Fukui, Japan

**Correspondence**: Taiyo Morikawa - taiyo1125@hotmail.co.jp

*Clinical and Translational Allergy* 2017, **7(Suppl 3)**:O02

**Introduction**: It is well known that T helper (Th) 2 cells and group 2 innate lymphoid cells (ILC2s) contribute to allergic diseases. However, their exact role and relationship in nasal allergic disorders is unclear. We sought to investigate the cooperation of Th2 cells and ILC2s in a mouse model of nasal allergic disorder.

**Methods**: To differentially activate Th2 cells and/or ILC2s in nasal mucosa, mice were intranasally administered ovalbumin (OVA) antigen, papain, an ILC2-activatior, or both for 2 weeks. Epithelial thickness and number of eosinophils in the nasal mucosa were evaluated at 24 h after the final challenge.

**Results**: Intranasal administration of OVA and papain preferentially activated Th2 cells and ILC2s, respectively, in the nose. Both OVA and papain increased the nasal epithelial thickness and number of eosinophils, and their coadministration significantly enhanced the symptoms. ILC2- and Rag2-deficient mice showed a partial decrease in OVA-plus-papain-induced nasal epithelial thickening and eosinophilia. Interleukin (IL)-33- and ST2-deficient mice showed decreased OVA-plus-papain-induced, but not OVA-alone-induced nasal epithelial thickening and eosinophilia. IL-5 induced eosinophilia only, but IL-13 contributed to both nasal epithelial thickening and eosinophilia.

**Conclusions**: IL-33/ST2-pathway-mediated ILC2 activation exacerbated additively Th2-cell-induced nasal type 2 inflammation. Furthermore, IL-13, but not IL-5, contributes to exacerbation of nasal type 2 inflammation.

**Keywords**: Nasal allergy, Th2 cells, Group 2 innate lymphoid cells, IL-33, IL-13

### O03 Allergen endotoxins induce non-IgE-mediated nasal hypersensitivity in mice via monocyte/macrophage-dependent pathway

#### Tomohiro Yoshimoto, Naruhito Iwasaki, Kazufumi Matsushita

##### Hyogo College of Medicine, Nishinomiya, Japan

**Correspondence**: Tomohiro Yoshimoto - tomo@hyo-med.ac.jp

*Clinical and Translational Allergy* 2017, **7(Suppl 3)**:O03

**Introduction**: Allergen-mediated cross-linking of IgE on mast cells/basophils is a well-recognized trigger for type-1 allergic diseases such as allergic rhinitis (AR). However, allergens may not be the only trigger for AR, and several allergic-like reactions are induced by non-IgE-mediated mechanisms. Here, we describe a novel non-IgE-mediated, endotoxin-triggered nasal type-1-hypersensitivity reaction in mice.

**Methods**: To investigate whether endotoxin affects sneezing responses, mice were intraperitoneally immunized with ovalbumin (OVA), and then nasally challenged with endotoxin-free or endotoxin-containing OVA. To investigate the role of T cells and mechanisms of the endotoxin-induced response, mice were adoptively transferred with in vitro differentiated OVA-specific Th2 cells, and then nasally challenged with endotoxin-free or endotoxin-containing OVA. Immediately after each nasal challenge, the frequency of sneezing was counted for 10 min. The mice were sacrificed 24 h after the final nasal challenge, and noses were dissected for analyzing infiltrating inflammatory cells.

**Results**: Endotoxin-containing, but not endotoxin-free, OVA elicited sneezing responses in mice independent from IgE-mediated signaling. OVA-specific Th2 cell adoptive transfer to mice demonstrated that local activation of antigen-specific Th2 cells was required for the response. The Toll-like receptor 4-MyD 88-signaling pathway was indispensable for endotoxin-containing OVA-elicited rhinitis. In addition, lipopolysaccharide directly triggered sneezing responses in OVA-specific Th2-transferred and nasally endotoxin-free OVA-primed mice. Although an antihistamine, diphenhydramine, suppressed sneezing responses, mast-cell/basophil-depleted mice had normal sneezing responses to endotoxin-containing OVA. Clodronate treatment abrogated endotoxin-containing OVA-elicited rhinitis, suggesting the involvement of monocytes/macrophages in this response.

**Conclusions**: Antigen-specific nasal activation of CD4^+^ T cells followed by endotoxin exposure induces mast cell/basophil-independent histamine release in the nose that elicits sneezing responses. Thus, environmental or nasal residential bacteria may exacerbate AR symptoms. In addition, this novel phenomenon might explain currently unknown mechanisms in non–IgE-mediated allergic disorders, such as non-IgE-mediated gastrointestinal food allergy in infants.

**Keywords**: Non-allergic rhinitis, Endotoxin, Histamine, T cells, Monocytes/macrophages

## Oral Abstract Session 2: Omics in Rhinology

### O04 Increased microbial abundance and decreased diversity in preschool children at risk for asthma

#### Tamar Smulders, Danielle Van Egmond, Kees Van Drunen, Marc Van Der Schee

##### AMC-UvA, Amsterdam, The Netherlands

**Correspondence**: Tamar Smulders - t.smulders@amc.uva.nl

*Clinical and Translational Allergy* 2017, **7(Suppl 3)**:O04

**Introduction**: Recent studies suggest that a specific respiratory microbiome is associated with an increased risk of viral infection and that in turn these infections influence the composition of the resident microbiota. A commonly found virus that causes infection in preschool children is *Rhinovirus* (RV), wheeze is an important symptom. Children with a symptomatic RV infection have an increased risk to develop persistent wheeze and asthma.

If children with a RV infection have a specific respiratory microbiome this may hold implications for early diagnosis of asthma and novel therapeutic approaches aimed at restoring microbial dysbiosis.

**Methods**: We aimed to study this by comparing the nasal microbiome of children with physician confirmed wheeze, during acute symptoms and after recovery, to symptomatic controls with non-wheezing respiratory illness and asymptomatic healthy controls. Specificity of our outcomes for RV was determined by matched analysis in RV negative (RV-) children. As part of the EUROPA-study children were visited within 8 h of exhibiting respiratory symptoms and again upon recovery for assessment of symptoms and nasal swab collection. Swabs were tested by qPCR for 14 respiratory viruses. Bacterial microbiota analysis was done by IS-pro, a 16S-23S PCR-based bacterial profiling technique. Relative abundance and Shannon diversity index were compared between groups.

**Results**: 160 pre-school children were included in the study. RV induced wheeze had the highest normalized total bacterial abundance (mean ± SEM; 0.95 ± 0.08) followed by symptomatic controls (0.77 ± 0.08) and asymptomatic controls (0.57 ± 0.09, p = 0.01). This increase was related to an upregulation of bacterial species belonging to the phyla of *Firmicutes* and *Bacteroidetes* of which the latter persisted after recovery from infection. None of the control subjects carried *Bacteroidetes*. Microbial diversity was significantly (p = 0.04) decreased in RV+ wheezing children (Median [IQR] 1.60 [0.97]) compared to RV− wheezing children (2.00 [0.75]).

**Conclusions**: We established an increased microbial abundance and decreased microbial diversity in children with *Rhinovirus* induced wheeze who are at an increased risk to develop asthma. For a subset of these children this was primarily attributed to shifts in various species belonging to the phylum of *Bacteroidetes*. Our findings hold potential implications for the early diagnosis of asthma and novel therapeutic approaches aimed at restoring microbial dysbiosis.

**Keywords**: Asthma, Respiratory Microbiome, Rhinovirus, Wheeze, IS-Pro

### O05 Proteomics of eosinophilic mucin

#### Achim Georg Beule

##### Klinik für Hals, Nasen- und Ohrenheilkunde, Universitätsklinikum Münster, Münster, Germany

**Correspondence**: Achim Georg Beule - AchimGeorg.Beule@ukmuenster.de

*Clinical and Translational Allergy* 2017, **7(Suppl 3)**:O05

**Introduction**: Eosinophilic mucin is a clinical relevant nasal secretion in patients with chronic rhinosinusitis (CRS) warning the surgeon of increased risks of recurrence of both nasal polyps and symptoms. While the pathophysiology leading to this thick, glue-like mucus is poorly understood, physicians are increasingly confronted to employ topical and or systemic strategies to avoid postoperative deterioration. Aim of this study was to determine a typical proteomics signature of eosinophilic mucin to improve our understanding of the underlying pathophysiology.

**Methods**: Nasal secretions were collected from 10 healthy volunteers and compared to intraoperatively collected specimens of eosinophilic mucin. Pooled samples were analyzed using 2D-Gel PAGE, MALDI-MS and IPA analysis. Gender contributions was balanced and topical medication as well as allergy as confounding factors excluded.

**Results**: 593 proteins were identified, of which 373 proteins were present in both pooled samples (mucin only 76, healthy lavage only 144 proteins). 303 out of 393 proteins were regulated in their expression. If a cut-off value of 1.5 was determined, 43 were induced and 40 were reprimed in eosinophilic mucin.

**Conclusions**: Based on our results, specific proteomic changes could be observed in eosinophilic mucin enabling further insight into the specific pathophysiology leading to deterioration in this subgroup of CRS patients.

## Poster Discussion Session 1: Rhinitis

### P01 Probiotics impregnated bedding covers in house dust mite allergic rhinitis patients: a pilot randomized controlled trial

#### Margot Berings^1^, Anton Jult^2^, Hanne Vermeulen^2^, Natalie De Ruyck^1^, Lara Derycke^1^, Hakan Ucar^3^, Philip Ghekiere^3^, Robin Temmerman^4^, John Ellis^5^, Claus Bachert^1^, Bart Lambrecht^6^, Melissa Dullaers^6^, Philippe Gevaert^6^

##### ^1^Upper Airways Research Laboratory, Ghent University, Ghent, Belgium; ^2^Ghent University, Ghent, Belgium; ^3^BekaertDeslee Innovation bvba, Waregem, Belgium; ^4^Chrisal NV, Lommel, Belgium; ^5^Devan Chemicals NV, Ronse, Belgium; ^6^Laboratory of Immunoregulation, VIB Inflammation Research Center, Ghent University, Ghent, Belgium

**Correspondence**: Margot Berings - margot.berings@ugent.be

*Clinical and Translational Allergy* 2017, **7(Suppl 3)**:P01

**Introduction**: House dust mite exposure is a major cause of allergy worldwide. As current evidence for existing house dust mite avoidance measures is low, new methods are being developed.

**Objectives**: To explore the effect of probiotics-impregnated bedding covers on symptoms and quality of life of patients with allergic rhinitis to house dust mite.

**Methods**: A double-blind, randomised, placebo-controlled, crossover trial was conducted at Ghent University Hospital, Belgium. The pilot trial included 20 adult patients with allergic rhinitis to house dust mite. The trial consisted of an 8-week period with untreated (placebo) covers and an 8-week period with probiotics-impregnated covers in random order, with a washout period in between of at least 4 weeks. Der p 1 concentrations were measured in dust samples collected from mattresses and pillows. Symptoms and quality of life were assessed through self-reported questionnaires.

**Product information**: The probiotics-based textile treatment contains five different probiotic and natural bacterial strains of Bacillus species (*Bacillus subtilis*, *Bacillus amyloliquefaciens* and *Bacillus pumilus*). The probiotics are incorporated in microcapsules, which are diffusely inserted in the textile. The probiotics remain non-active in the closed microcapsules, until upon friction forces a small number of the microcapsules rupture and release their probiotic bacteria.

**Results**: There was a comparable and significant reduction of Der p 1 levels with both the probiotics-impregnated covers and the untreated covers. Several symptom and quality of life scores improved significantly with the probiotics-impregnated covers, whereas no significant changes were observed with the untreated covers. The effects of the probiotics-impregnated covers on symptoms and quality of life scores however were not significant compared to the placebo covers (except for a subscore ‘*NRQLQ sleeptime’)*.

**Conclusions**: This pilot study suggests that probiotics-impregnated bedding covers may improve symptoms and quality of life of patients with allergic rhinitis to house dust mite. Although the effects of the probiotics-impregnated covers were not significant compared to the untreated covers, these findings are promising and warrant a future large-scale clinical trial.

*Prior to patient enrolment, the trial was registered at clinical.trials.gov (NCT01997606).*

**Keywords**: Allergy, Rhinitis, House dust mite, Probiotics, Avoidance

### P02 Reliable mite-specific IgE testing in nasal secretions by means of allergen microarray

#### Stefania Arasi^1^, Margot Berings^2^, Serena Perna^1^, Natalie De Ruyck^2^, Yvonne Resch^3^, Christian Lupinek^3^, Kuan-Wei Chen^3^, Susanne Vrtala^3^, Rudolf Valenta^3^, Paolo Maria Matricardi^1^, Philippe Gevaert^2^

##### ^1^Department of Pediatric Pneumology and Immunology, Charité Medical School, Berlin, Germany; ^2^Upper Airway Research Laboratory, Ghent University, Ghent, Belgium; ^3^Medical University of Vienna, Vienna, Austria

**Correspondence**: Stefania Arasi - stefania.arasi@yahoo.it

*Clinical and Translational Allergy* 2017, **7(Suppl 3)**:P02

**Introduction**: In nasal secretions (NS) IgE is present at very low concentrations and difficult to detect with standard methods.

To evaluate the performance of a customized microarray chip (Thermo Fisher Scientific, TFS, Uppsala, Sweden) derived from the new ImmunoCAP ISAC 103™ for simultaneous detection of IgE to 13 components of *Dermatophagoides pteronyssinus* (D.pt.) and 2 of *Dermatophagoides farinae* (D.fa.) in serum and NS.

**Methods**: NS were collected with both Filter Disks (FD) and Sinus Packs (SP) in 30 adult patients with allergic rhinitis to HDM and 29 adult non-allergic controls. NS samples were diluted 1:20 for FD and 1:10 for SP. Specific IgE to 13 D.pt. and 2 D.fa. components (Der p 1, rDer p 2, Der p 4, rDer p 5, rDer p 7, rDer p 10, rDer p 11, rDer p 14, rDer p 15, rDer p 18, rDer p 21, rDer p 23, clone 16; Der f 1 and Der f 2) were measured with the microarray both in serum (cut-off ≥ 0.10 ISAC standardized units [ISU]) and in NS. In NS the cut-off for IgE positivity needs to be determined. Hence, several analytical cut-off levels were tested (final cut-off reported below). Sensitivity and specificity of IgE tests in NS were calculated using the results of IgE tests in serum as a reference.

**Results**: With the microarray, the best balance of sensitivity and specificity was achieved with a cut-off of ≥0.03 ISU in the diluted NS. With this cut-off, nasal IgE to major allergen molecules (nDer p 1, nDer f 1, rDer p 2, rDer f 2, rDer p 23) identified the mite-allergic patients with 90% sensitivity and 100% specificity.

Among the mite allergic subjects, prevalences of IgE positivity to 13 of the 15 examined components were similar in serum and NS: 83–97% for Der p 2 and Der f 2; 70–87% for Der p 23; 20–33% for Der p 5 and Der p 7; and 0–23% for all others. IgE to Der p 1 and Der f 1 were detected more frequent in serum (80–83%) than in NS (37–60%).

Among the non-allergic controls, no IgE to the components was detected in sera and NS, with the exception of IgE to Der p 5 that was detected (= 0.03 ISU) in 4 controls but only in their SP samples.

**Conclusions**: An ISAC-derived microarray technique detected nasal IgE to major allergen molecules D.pt. and D.fa (nDer p 1, nDer f 1, rDer p 2, rDer f 2, rDer p 23) identifying the mite-allergic patients with 90% sensitivity and 100% specificity when referred to serum. These promising findings need confirmation in larger studies (Table 1).

**Table:** Table 1 Prediction of house dust mite (HDM) allergy and serum IgE responses by testing IgE in nasal secretions in 30 HDM allergic patients and 29 healthy controls

	Sensitivity		Specificity		PPV		NPV		Accuracy	LR+	LR−
	%	(95% CI)^†^	%	(95% CI)^†^	%	(95% CI)^†^	%	(95% CI)^†^	%		
Filter disk											
ANY major molecules°	90	(73–98)	100	(83–100)	100	(82–100)	91	(75–98)	95	∞	0.1
*nDer p 1*	46	(26–66)	100	(85–100)	100	(62–100)	73	(58–85)	78	∞	0.5
*nDer f 1*	68	(46–85)	100	(85–100)	100	(73–100)	81	(66–91)	86	∞	0.3
*rDer p 2*	90	(73–98)	100	(83–100)	100	(81–100)	91	(76–98)	95	∞	0.1
*rDer f 2*	90	(73–98)	100	(83–100)	100	(81–100)	91	(76–98)	95	∞	0.1
*rDer p 23*	81	(61–93)	100	(85–100)	100	(77–100)	87	(72–96)	92	∞	0.2
OTHER molecules											
*rDer p 4*	0	(0–53)	98	(90–100)	0	(0–99)	88	(77–95)	86	0.0	1.0
*rDer p 5*	80	(44–97)	100	(89–100)	100	(52–100)	96	(87–100)	97	∞	0.2
*rDer p 7*	78	(40–97)	100	(90–100)	100	(47–100)	96	(87–100)	97	∞	0.2
*rDer p 21*	57	(18–90)	100	(90–100)	100	(28–100)	95	(85–99)	95	∞	0.4
ALL molecules^¥^	71	(63–77)	100	(99–100)	98	(93–100)	93	(91–95)	94	167.4	0.3
Sinus Pack											
ANY major molecules°	87	(69–96)	100	(83–100)	100	(81–100)	88	(72–97)	93	∞	0.1
*nDer p 1*	58	(37–78)	100	(85–100)	100	(68–100)	78	(63–89)	83	∞	0.4
*nDer f 1*	48	(28–69)	100	(85–100)	100	(64–100)	72	(57–84)	78	∞	0.5
*rDer p 2*	86	(68–96)	100	(83–100)	100	(80–100)	88	(73–97)	93	∞	0.1
*rDer f 2*	86	(68–96)	100	(83–100)	100	(80–100)	88	(73–97)	93	∞	0.1
*rDer p 23*	81	(61–93)	100	(85–100)	100	(77–100)	87	(72–96)	92	∞	0.2
OTHER molecules											
*rDer p 4*	14	(0–58)	98	(90–100)	50	(1–99)	89	(78–96)	88	7.4	0.9
*rDer p 5*	70	(35–93)	90	(78–97)	58	(28–85)	94	(82–99)	86	6.9	0.3
*rDer p 7*	67	(30–93)	100	(90–100)	100	(42–100)	94	(84–99)	95	∞	0.3
*rDer p 21*	57	(18–90)	100	(90–100)	100	(28–100)	95	(85–99)	95	∞	0.4
ALL molecules^¥^	68	(60–75)	99	(98–100)	95	(90–98)	93	(91–94)	94	80.3	0.3

^†^ Exact binomial confidence limits (95% CI) for test sensitivity, specificity, PPV (positive predictive value); NPV (negative predictive value), LR + (positive likelihood ratio), LR- (negative likelihood ratio)

° Outcomes referred to at least one of the major allergen molecules (nDer p 1, nDer f 1, rDer p 2, rDer f 2, rDer p 23)

^¥^ Including outcomes of rDer p 10, rDer p 11, rDer p 14, rDer p 15, Clone 16, rDer p 18, all characterized by a low positive sample size (n < 5)

**Keywords**: Allergen Molecules, Allergic Rhinitis, House Dust Mite Allergy, Immunoglobulin E, Nasal Secretions

### P03 Correlation between rhinomanometry and spirometry parameters in 971 adults

#### Ivânia Gonçalves^1^, Tiago Jacinto^2^, Rita Amaral^3^, Ana M. Pereira^3^, Luís M. Araújo^4^, Mariana Couto^1^, João A. Fonseca^5^

##### ^1^CUF Porto - Instituto & Hospital, Porto, Portugal; ^2^CUF Porto - Instituto & Hospital; CINTESIS - Center for Health Technology and Services Research; ESS - IPP - School of Health; Polytechnic of Porto, Porto, Portugal; ^3^CUF Porto - Instituto & Hospital; CINTESIS - Center for Health Technology and Services Research, Porto, Portugal; ^4^CUF Porto - Instituto & Hospital; Allergy Unit, FMUP - Faculty of Medicine, University of Porto, Porto, Portugal; ^5^CUF Porto - Instituto & Hospital; CINTESIS - Center for Health Technology and Services Research; MEDCIDES - Department of Health Information and Decision Sciences, Faculty of Medicine, University of Porto, Porto, Portugal

**Correspondence**: Ivânia Gonçalves - ivania.m.g@gmail.com

*Clinical and Translational Allergy* 2017, **7(Suppl 3)**:P03

**Introduction**: There is a lack of published studies about the association between rhinomanometry and spirometry results. Some studies have shown a moderate correlation between spirometry parameters and other nasal objective measures such as Peak Nasal Inspiratory Flow (PNIF). We aimed to study the correlation between rhinomanometry and spirometry parameters.

**Methods**: We included all adults (age ≥ 18 years) who performed rhinomanometry and spirometry consecutively on the same day, at CUF Porto - Instituto and Hospital from November 2010 to July 2016. When more than one rhinomanometry was performed, only the first one was included in the analysis. We included gender, age, height, rhinomanometry parameters (inspiratory and expiratory total nasal airflow, inspiratory (*RAARi*) and expiratory (*RAARe*) mean airflow resistance at a sample pressure of 150 Pa and side ratio) and spirometric parameters (forced vital capacity (FVC), forced expiratory volume in 1 s (FEV_1_), mid-flow rate/forced expiratory flow at 25–75% of FVC (FEF25-75) and FEV_1_/FVC). Pearson’s correlation was used to evaluate unadjusted correlations and partial correlations were used to adjust parameters for age, gender and height.

**Results**: A total of 971 adults were included, 623 (64%) females, with a mean (sd) height of 166.0 (9.0) cm and age of 38.3 (14.1) years (min–max: 18–80). Correlations between spirometry and rhinomanometry variables are presented in Table 1. The correlations between FEV_1_/FVC and either inspiratory total nasal airflow or mean RAARi, were the only ones with statistical significance (r = −0.083, p = 0.016 and r = 0.011, p = 0.039, respectively). After adjusting for age, gender and height, no statistically significant associations were found between the parameters.

**Table:** Table 1 Results

	FVC (%)	*p*	FEV1 (%)	*p*	FEV1/FVC (%)	*p*	MEF25-75 (%)	*p*
Insp total nasal airflow (ml/s)	−0.027	0.575	0.017	0.615	**−0.083**	**0.016**	−0.008	0.808
Exp total nasal airflow (ml/s)	0.032	0.359	0.052	0.131	−0.055	0.116	0.047	0.173
Mean RAARi (kPa*s/L)	0.092	0.880	−0.027	0.424	**0.011**	**0.039**	−0.047	0.160
Mean RAARe (kPa*s/L)	−0.006	0.862	−0.044	0.186	−0.012	0.731	−0.057	0.087
Side ratio	−0.032	0.341	−0.046	0.165	−0.023	0.495	−0.023	0.487

Bold formatting equals the values with statistical significance

**Conclusions**: Rhinomanometry and spirometry parameters were not significantly correlated after adjustment to confounders, which suggests that rhinomanometry measurements are not influenced by respiratory capacity measured with spirometry, contrary to PNIF. This may be advantageous, especially in patients with low respiratory functional capacity.

**Keywords**: Rhinomanometry, Spirometry

### P04 Profile of patients with persistent allergic rhinitis prescribed MP-AzeFlu^®^* In routine clinical practice: pooled data from Austria, Ireland and Sweden

#### Par Stjarne^1^, Ranbir Kaulsay^2^, Wolfgang Pohl^3^

##### ^1^Karolinska Institute, Stockholm, Sweden; ^2^Clontarf Clinic, Dublin, Ireland; ^3^Karl Landsteiner Institut fur Experimentelle und Klinische Pneumologie, Vienna, Austria

**Correspondence**: Par Stjarne - par.stjarne@karolinska.se

*Clinical and Translational Allergy* 2017, **7(Suppl 3)**:P04

**Introduction**: The aims of this study were (i) to characterise patients with persistent allergic rhinitis (PER) prescribed Meda Pharma’s AzeFlu (MP-AzeFlu; a novel formulation of azelastine hydrochloride, fluticasone propionate and excipients in a single spray) in real-life in Austria, Ireland and Sweden and (ii) to quantify the personal symptomatic burden of PER in these countries prior to MP-AzeFlu prescription.

**Methods**: 428 patients (≥12 years old) with moderate-to-severe PER were recruited into 3, prospective, non-interventional studies carried out in Austria (n = 214), Ireland (n = 53) and Sweden (n = 161). MP-AzeFlu was prescribed according to label. Information was gathered on patient demographics, AR phenotype, allergen sensitization, symptomatology, previous AR treatments in the last year (prior to MP-AzeFlu prescription) and reason for MP-AzeFlu prescription. Data for all countries are pooled.

**Results**: Classified traditionally, slightly more patients had both seasonal AR (SAR) and perennial AR (PAR) (n = 254; 59.3%) vs. PAR alone (n = 174; 40.7%). Sensitization to house dust mite predominated (n = 261; 61.0%), followed by animal dander, and at least 50.5% (n = 216) were poly-sensitized. Prior to MP-AzeFlu prescription patients reported troublesome symptoms (n = 268; 62.6%), impairment of daily activities (n = 238; 55.6%), sleep disturbance (n = 235; 54.9) and impairment of school/work (n = 177; 41.4%). Congestion was considered the most bothersome symptom by most patients (n = 254; 59.3%). The most frequent reason for MP-AzeFlu prescription was that other therapies were not sufficient in the past (n = 299; 69.9%) or not sufficient to treat acute symptoms (n = 87; 20.3%). Most of these PER patients were previously treated with oral antihistamines (n = 274; 64.0%), intranasal corticosteroids (n = 236; 55.1%) or intranasal anti-histamines (n = 97; 22.7%). 59.3% (n = 254) of patients reported using ≥2 AR therapies in the past year, but 9.6% (n = 41) reported using no AR therapy at all.

**Conclusions**: Many patients in Europe live with uncontrolled persistent disease despite treatment with mono- and multiple therapies. A more effective treatment option, like MP-AzeFlu, should improve AR control and reduce the number of patients requiring immunotherapy.

**Keywords**: MP-AzeFlu, Dymista, Azelastine, Fluticasone Propionate

*MP-AzeFlu, a registered trademark of Meda AB, is marketed in the U.S. as Dymista^®^, a registered trademark of Meda Pharma Inc., both Mylan Companies.

### P05 Measurement of nasal specific IgE in patients with local allergic rhinitis

#### Paloma Campo^1^, Ibon Eguiluz^2^, Carmen Rondon^3^, Maria Carmen Plaza^4^, Ana Maria Prieto^5^, Luisa Galindo^1^, Cristobalina Mayorga^2^, Miguel Blanca^4^, Maria Jose Torres^5^

##### ^1^Allergy Unit, Malaga, Spain; ^2^IBIMA, Malaga, Spain; ^3^Research Laboratory, Malaga, Spain; ^4^Regional University Hospital of Malaga, Malaga, Spain; ^5^UMA, Malaga, Spain

**Correspondence**: Ibon Eguiluz - iboneguiluz@gmail.com

*Clinical and Translational Allergy* 2017, **7(Suppl 3)**:P05

**Introduction**: Prior methods used for measuring nasal specific IgE (NsIgE) in local allergic rhinitis (LAR) have shown a variable sensitivity: 22% for *D. Pteronyssinus* (DP) using the Greiff/Grünberg method and lower with Naclerio method. In this study a novel method of detection of NsIgE in patients with confirmed LAR to DP was evaluated.

**Methods**: Sixteen LAR (positive nasal allergen provocation test to DP (NAPT-DP), negative skin testing/sIgE to DP), 10 allergic rhinitis (AR) as positive control (positive NAPT-DP and skin testing/sIgE to DP), and 12 healthy controls as negative control (negative NAPT-DP and skin testing/sIgE to DP) were recruited. DP-ImmunoCAP^®^ solid phase was applied directly in the lower turbinate of each nostril for 10 min before and 24 h after NAPT-DP and analyzed following the manufacturer´s instructions. ROC curves were performed to obtain the optimal cut-off point of nasal sIgE value to calculate sensitivity (S) and specificity (SP), and outcomes were compared with NAPT-DP result (gold standard test). Study was approved by local ethics committee.

**Results**: All LAR and AR subjects had a positive response to NAPT-DP, and none in the healthy control group. At 24 h after NAPT-DP, mean NsIgE values were 0.119 kU/L in LAR, 1.600 kU/L in AR and 0.115 kU/L in healthy controls. ROC curves using NsIgE values obtained 24 h after NAPT-DP were performed. In LAR subjects, the area under the curve (AUC) was 0.7277, p = 0.0054. The optimal cut-off point to discriminate LAR subjects from controls was 0.135 kU/L, obtaining a S = 20.31% and SP = 88.09%. In AR (positive control group) the AUC was 0.9798, p ≤ 0.0001, and the optimal cut-off point was 0.170 kU/L with S = 95% and SP = 100%.

**Conclusions**: Measurement of NsIgE by direct application of DP-ImmunoCAP^®^ in LAR shows similar sensitivity to other methods and good specificity, with the advantage of being non-invasive, easier to perform and faster. Funded by Institute of Health “Carlos III” (Ministry of Economy and Competitiveness) RETICS ARADyAL (RD16/0006/0001), FIS PI14/00864 and Consejería de Salud PI-0346-2016.

**Keywords**: IgE, Local Allergic Rhinitis, Nasal

### P06 Patients with persistent allergic rhinitis get a better night’s sleep on MP-AzeFlu^®^*: individual and pooled data from Austria, Ireland and Sweden

#### Ranbir Kaulsay^1^, Wolfgang Pohl^2^, Par Stjarne^3^

##### ^1^Clontarf Clinic, Dublin, Ireland; ^2^Karl Landsteiner Institut fur Experimentelle und Klinische Pneumologie, Vienna, Austria; ^3^Karolinska Institute, Stockholm, Sweden

**Correspondence**: Ranbir Kaulsay - ranbir@clontarftclinic.com

*Clinical and Translational Allergy* 2017, **7(Suppl 3)**:P06

**Introduction**: Most allergic rhinitis (AR) patients attending clinic have moderate/severe persistent disease and frequently report reduced sleep quality. Meda Pharma’s AzeFlu (MP-AzeFlu) comprises intranasal azelastine hydrochloride, fluticasone propionate and a novel formulation, in a single device. Its real-life effectiveness has been established in AR during 14 days. However, its impact on sleep quality is unknown. This study assessed the impact of MP-AzeFlu on sleep quality when used in routine clinical practice by patients with persistent AR (PER).

**Methods**: 428 patients (≥12 years old) with moderate-to-severe PER were recruited into 3, prospective, non-interventional studies carried out in Austria (n = 214), Ireland (n = 53) and Sweden (n = 161). MP-AzeFlu was prescribed according to its summary of product characteristics. Patients assessed their sleep quality (7-days reflective) on days 7, 14, 21, 28, 35 and 42 using a 5-point scale from ‘very good’ to ‘very bad’.

**Results**: Many patients in each country reported sleep disturbance prior to MP-AzeFlu prescription: n = 112 (52.3%) in Austria; n = 41 (77.4%) in Ireland; n = 82 (50.9%) in Sweden. MP-AzeFlu treatment (1 spray/nostril bd; daily doses:AZE = 548 μg;FP = 200 μg) was associated with improved sleep quality, evidenced by an increase in the proportion of patients reporting ‘very good’ and ‘good’ quality sleep in the first 28 days of treatment, and a corresponding reduction in the proportion of patients reporting ‘bad’ or ‘very bad’ sleep quality. Sleep quality improved at each assessed time point (Table 1). Improved sleep quality occurred irrespective of phenotype (when classified traditionally)—in those with perennial AR (PAR) only and in those with both PAR & seasonal AR.

**Table:** Table 1 Results

	Austria^†^ (n ≤ 214)		Ireland (n ≤ 53)		Sweden^†^ (n ≤ 161)		Pooled^†^ (n ≤ 428)	
	Day 0 (%)	Day 28 (%)	Day 0 (%)	Day 28 (%)	Day 0 (%)	Day 28 (%)	Day 0 (%)	Day 28 (%)
Very good	2.4	35.3	0.0	24.5	3.7	15.7	2.6	26.5
Good	25.1	44.6	24.5	50.9	28.6	44.4	26.4	46.0
Fair	36.5	17.3	32.1	15.1	34.2	25.9	35.1	20.1
Bad	28.0	2.2	32.1	5.7	27.3	12.0	28.3	6.4
Very bad	8.1	0.7	9.4	0.0	6.2	1.9	7.5	1.0

^†^  % without missing values

*MP-AzeFlu, a registered trademark of Meda AB, is marketed in the U.S. as Dymista^®^, a registered trademark of Meda Pharma Inc., both Mylan Companies.

Character count: 2352 (limit = 2500).

**Conclusions**: MP-AzeFlu improves sleep quality in patients with moderate-to-severe PER in a real-world pan-European setting.

**Keywords**: Dymista, MP-AzeFlu, Azelastine, Fluticasone, Rhinitis

### P07 Facial infrared thermography in the assessment of nasal provocation test

#### Magdalena Herknerova

##### Hospital Na Homolce, Prague, Czech Republic

**Correspondence**: Magdalena Herknerova - MagdalenaH@centrum.cz

*Clinical and Translational Allergy* 2017, **7(Suppl 3)**:P07

**Introduction**: Infrared thermography is a diagnostic tool in more medical disciplines. It detects temperature changes in the infra-red spectrum range and was originally designed to test the heating qualities of buildings. Potential benefits in allergology and ENT are not widely evaluated. We asked if this method can give us an objective information about a patient during nasal provocation test.

**Methods**: As it is difficult to detect always the same point in the face during repeating measurements, a bounding box to detect the face of a patient was designed.

Forehead, nasal, cheek and chin facial areas were defined.

**Results**: We present one example of our software in the evaluation of facial infrared thermography. This method detects temperature changes in different areas of the face. The nasal area was the most sensitive one in the detection of temperature change during nasal provocation test followed by the cheek area.

**Conclusions**: Facial infrared thermography might be- besides other important parametres like nasal symptome score and active anterior rhinomanometry- an objective, non- invasive and feasible tool in the assessment of allergic response during nasal provocation test.

**Keywords**: Infrared Thermography, Nasal Provocation, Bounding Box

### P08 Intranasal condyloma acuminatum with malignant transformation

#### Tengchin Wang^1^, Chiejun Wu^2^

##### ^1^Department of Otolaryngology, Tainan Municipal Hospital, Tainan City, Taiwan; ^2^Department of Pathology, Tainan Municipal Hospital, Tainan City, Taiwan

**Correspondence**: Tengchin Wang - tengchin27@hotmail.com

*Clinical and Translational Allergy* 2017, **7(Suppl 3)**:P08

**Introduction**: Condyloma acuminatum is a venereal diasese transmitted by the human papillomavirus (HPV). Generally, it is a benign entity but carcinomatous change has been reported in anogenital area. The malignant transformation is associated with the immunocompromised status,especially HIV. Condyloma acuminatum is uncommonly identified in the nasal cavity, the malignant transformation is extremely rare.

**Methods**: A mid-aged patient with diabetes mellitus and psoriasis had suffered from progressive right nasal obstruction with epistaxis for six months. Physical examination revealed a cauliflower-like lesion over the right nasal vestibule, expanding to the septum. Due to this patient had history of penile condyloma acuminatum, biopsy was done and sent hc2 high-risk-HPV DNA testing. The results was compatible with condyloma acuminatum and negative for high-risk-HPV infection.

**Results**: Eradication surgery was performed. Necrotic tissue with pus content was buried in the lesion. This mass was completely excised eventually. The formal histopathology reported condyloma acuminatum with focal invasive squamous cell carcinoma. We suggested this patient receiving adjuvant radiotherapy or re-operation because there was no planed safe margin in advance, and the examinations for HIV and syphilis were also advised. But the patient refused and lost of follow-up thereafter.

**Conclusions**: Human papillomavirus types 16 and 18 are found in up to 90% of patients with cervical carcinoma, however HPV type 6 and HPV type 11 are the main factor in developing giant condyloma acuminatum, which is reported 56% incidence of malignant transformation. Abscesses and fistulas are more common in lesions as described in our case. Immunosuppression, coexisting HIV infection, and unhygienic conditions play a role in malignant transformation, therefore AIDS togethers with other venereal diseases should be examined. Electrocautery or laser total excision could be applied for treatment. Non-resectable lesions, radiotherapy could be applied either alone or along with chemotherapy. In conclusion, condyloma acuminatum with invasive squamous cell carcinoma is rarely found in the nasal cavity, more cases should be obtained for more comprehensive understanding.

**Keywords**: Condyloma Acuminatum, Immunocompromised

### P09 Rhinitis: classification and diagnosis algorithm proposal

#### Jonathan Kilimajer

##### Subiza Asthma and Allergy Center, Madrid, Spain

**Correspondence**: Jonathan Kilimajer - jkilimajer@hotmail.com

*Clinical and Translational Allergy* 2017, **7(Suppl 3)**:P09

**Introduction**: One of the most common problems presenting among physicians are rhinitis symptoms. Challenges in the diagnosis results from the factor that differential diagnosis is extensive and sometimes symptoms of allergic, nonallergic, and mixed rhinitis are often indistinguishable. Searching in the literature there is a clear consensus on the classification but is difficult to found one that include and ordered all the different etiologies. It is also difficult to found an specific diagnosis algorithm for these diseases.

**Methods**: After checking over and try to reunite different consensus made in allergy an ENT for classification and diagnosis of rhinitis we propose Tables 1, 2 with both of these sections.

**Results**

**Table:**
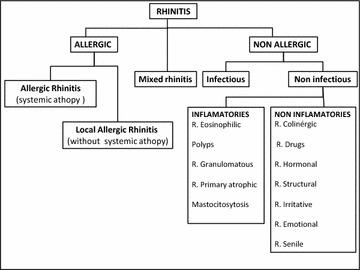
Table 1 Classification of Rhinitis

**Table:**
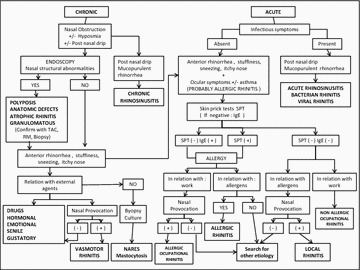
Table 2 Diagnosis algorithm for Rhinitis

**Conclusions**: When approaching rhinitis, diagnostic challenge is to determine the etiology, specifically whether it is allergic, nonallergic, or perhaps an overlap of both conditions.

With these proposal tables it could be more easy to differentiate and lead to a correct diagnosis.

### P10 Real life effectiveness of MP-AzeFlu^®^* in persistent allergic rhinitis, assessed by visual analogue scale and endoscopy: pooled data from Austria, Ireland and Sweden

#### Wolfgang Pohl^1^, Ranbir Kaulsay^2^, Par Stjarne^3^

##### ^1^Karl Landsteiner Institut fur Experimentelle und Klinische Pneumologie, Vienna, Austria; ^2^Clontarf Clinic, Dublin, Ireland; ^3^Karolinska Institute, Stockholm, Sweden

**Correspondence**: Par Stjarne - par.stjarne@karolinska.se

*Clinical and Translational Allergy* 2017, **7(Suppl 3)**:P10

**Introduction**: Most allergic rhinitis (AR) patients attending clinic have moderate/severe persistent disease. Meda Pharma’s AzeFlu (MP-AzeFlu) comprises intranasal azelastine hydrochloride, fluticasone propionate and a novel formulation, in a single device. Its real-life effectiveness has been established in AR during 14 days, but its effectiveness in those with persistent AR (PER) over the longer term is unknown. We assessed the effectiveness of MP-AzeFlu in routine clinical practice in PER patients, using the ARIA-endorsed language of AR control (i.e. visual analogue scale (VAS)). A VAS score cut-off of 50 mm is recommended to assess control and guide treatment decisions.

**Methods**: 428 patients (≥12 yrs old) with moderate/severe PER were recruited into 3, prospective, non-interventional studies in Austria (n = 214), Ireland (n = 53) and Sweden (n = 161). MP-AzeFlu was prescribed according to label. Patients assessed symptom severity using a VAS from 0 mm (not at all bothersome) to 100 mm (very bothersome) in the AM prior to MP-AzeFlu use, on Days 0, 1, 3, 7, 14, 21, 28, 35 and 42. An endoscopy was performed in the Irish study on Days 0 and 28. Symptoms of ‘oedema’, ‘discharge’ and ‘redness’ were scored on a 3-point scale for both nostrils (max score = 12).

**Results**: Patients treated with MP-AzeFlu (1 spray/nostril bd; daily doses: AZE = 548 μg;FP = 200 μg) had a VAS score reduction from 59.0 mm (SD 25.0) at Day 0 to 28.8 mm (SD 23.2) at Day 28 (p < 0.0001) and 23.2 mm (SD 21.5) on Day 42 (p < 0.0001), a reduction of 35.8 mm. This reduction was rapid, with statistical significance vs baseline noted from Day 1 (p < 0.0001), and was consistent irrespective of phenotype, patient age, and disease severity. On average MP-AzeFlu-treated patients achieved the 50 mm VAS score cut-off before Day 3. Total endoscopy score reduced from 7.5 (SD 3.1) at baseline to 3.5 (SD 2.5) at Day 28. The % of patients with severe oedema reduced from 53.1% at baseline to 3.8% at Day 28. A similar reduction in the incidence of thick/mucusy discharge (28.3–4.8%) and severe redness (34.9–0%) was also observed.

**Conclusions**: MP-AzeFlu provides effective and rapid PER control in a real-world pan-European setting assessed by VAS. Symptom improvement was noted from Day 1, sustained for 42 days and was associated with improvement in mucosal appearance after 28 days.

**Keywords**: MP-AzeFlu, Dymista, Azelastine, Fluticasone

*MP-AzeFlu, a registered trademark of Meda AB, is marketed in the U.S. as Dymista^®^, a registered trademark of Meda Pharma Inc., both Mylan Companies.

### P11 Overadditive effects of MP-AzeFlu* on antiinflammatory genes and inhibition of proinflammatory mediators compared to Fluticasone Propionate and Azelastine in Sinonasal Fibroblasts

#### Laura Pujols^1^, Jordi Roca-Ferrer^1^, Borja Callejas^1^, Mireya Fuentes-Prado^1^, Maria Perez-Gonzalez^1^, Isam Alobid^2^, Antonio Valero^2^, Cesar Picado^2^, Ruth Murray^3^, Joaquim Mullol^2^

##### ^1^IDIBAPS & CIBERES, Barcelona, Spain; ^2^IDIBAPS, CIBERES & University of Barcelona Hospital Clinic, Barcelona, Spain; ^3^Medscript, Dundalk, Ireland

**Correspondence**: Laura Pujols - lpujols@clinic.cat

*Clinical and Translational Allergy* 2017, **7(Suppl 3)**:P11

**Introduction**: MP-AzeFlu*, a novel intranasal formulation of azelastine hydrochloride (AZE) and fluticasone propionate (FP), has demonstrated superior clinical effects compared to these drugs in monotherapy in patients with allergic rhinitis and chronic rhinitis. MP-AzeFlu has previously shown greater antiinflammatory potency than FP and AZE in an in vitro model of eosinophilic inflammation. Our aim was to compare the effect of MP-AzeFlu, FP, and AZE on antiinflammatory gene transactivation and proinflammatory mediator inhibition in sinonasal cultured fibroblasts from nasal mucosa (NM) and chronic rhinosinusitis with nasal polyps (CRSwNP).

**Methods**: NM and CRSwNP fibroblast cultures (n = 6) were incubated with serial dilutions of MP-AzeFlu (1:10^2^–1:10^4^) or FP or AZE (equivalent dilutions) for 2 to 24 h with/without 1 ng/ml IL-1b. Glucocorticoid-induced leucine zipper (GILZ), mitogen-activated protein kinase phosphatase-1 (MKP-1), and COX-2 gene (RT-PCR) and IL-8 and GM-CSF protein (ELISA) expression was analysed. Results are expressed as fold-increase or % inhibition over control (mean ± SEM).

**Results**: MP-AzeFlu and FP (all dilutions, *P* < 0.05) and AZE (1:10^2^, *P* < 0.05) increased GILZ and MKP-1 gene expression in NM and CRSwNP fibroblasts. MP-AzeFlu (1:10^2^) had an overadditive effect on GILZ expression vs FP or AZE and on MKP-1 expression vs AZE (*P* < 0.05) (Table 1). MP-AzeFlu and FP provoked a similar inhibition of IL-1b-induced COX-2, IL-8, and GM-CSF in NM and CRSwNP fibroblasts (all dilutions, *P* < 0.05) but greater than AZE, which had no inhibitory effect.

**Table:** Table 1 Results

	NM fibroblasts		CRSwNP fibroblasts	
	2 h	6 h	2 h	6 h
*GILZ (fold increase)*				
MP-AzeFlu* (1:10^2^)	11.2 ± 1.1	36.5 ± 7.9	14.7 ± 4.5	31.4 ± 9.2
FP (1:10^2^)	9.4 ± 1.3^†^	19.8 ± 2.4^†^	13.9 ± 3.8	15.3 ± 4.5^†^
AZE (1:10^2^)	1.8 ± 0.1^†^	7.9 ± 2.8^†^	2.5 ± 0.8^†^	2.8 ± 0.9^†^
*MKP*-*1 (fold increase)*				
MP-AzeFlu* (1:10^2^)	6.5 ± 1.4	5.4 ± 1.7	7.2 ± 2.1	6.2 ± 2.4
FP (1:10^2^)	5.4 ± 0.6	4.7 ± 1.0	5.7 ± 1.5	4.8 ± 1.6
AZE (1:10^2^)	2.1 ± 0.3^†^	1.8 ± 0.6^†^	2.9 ± 1.0^†^	1.9 ± 1.3^†^

	COX-2	IL-8	GM-CSF	COX-2	IL-8	GM-CSF
*Proinflammatory markers (% inhibition vs control at 24* *h)*						
MP-AzeFlu (1:10^2^)	72.0 ± 7.1	61.5 ± 5.5	80.0 ± 5.7	91.3 ± 0.5	50.4 ± 4.3	85.6 ± 4.0
FP (1:10^2^)	93 ± 2.4	69.1 ± 3.2	81.4 ± 4.8	96.8 ± 0.7	61.3 ± 5.6	87.5 ± 3.7

This study has been sponsored by a research grant form MYLAN – MEDA

^†^ *p* < 0.05 vs MP-AzeFlu*

* Dymista

**Conclusions**: The superior clinical effect of MP-AzeFlu compared to corticosteroid or antihistamine monotherapy may be related to the overadditive induction of antiinflammatory genes and the inhibition of proinflammatory mediators. Our findings reveal some molecular basis for the therapeutic benefit of MP-AzeFlu in rhinitis and potentially in CRSwNP.

**Keywords**: MP-AzeFlu, Azelastine, Fluticasone Propionate, GILZ

## Oral Abstract Session 3: Chronic Rhinosinusitis – mechanisms

### O06 TLR4 mediates epithelial barrier dysfunction by Staphylococcus Aureus Enterotoxin B in CRSwNP

#### Brecht Steelant, Katleen Martens, Guy Boeckxstaens, Sven F. Seys, Peter W. Hellings

##### KU Leuven, Leuven, Belgium

**Correspondence**: Brecht Steelant - brecht.steelant@kuleuven.be

*Clinical and Translational Allergy* 2017, **7(Suppl 3)**:O06

**Introduction**: Epithelial barrier dysfunction plays a role in the pathophysiology of chronic rhinosinusitis with nasal polyps (CRSwNP). Toll-like receptors (TLRs) are thought to regulate epithelial barrier integrity, though its function has not been studied in CRSwNP thus far. The aim of this study was to investigate if TLR2 and TLR4 signaling is involved in regulating barrier function in CRSwNP.

**Methods**: Primary nasal epithelial cells from controls and CRSwNP patients (both n = 5) were isolated and grown at air–liquid interface on transwell inserts for 21 days. Epithelial integrity was evaluated by measuring transepithelial electrical resistance (TER) together with the expression of occludin and zonula occludens 1. TLR2 and TLR4 expression was evaluated using qRT-PCR. Primary nasal epithelial cells were stimulated with *Staphylococcus aureus* enterotoxin B (SEB) for 4 h and TER was evaluated. *In vitro,* TLR2 and TLR4 signaling was blocked to evaluate the contribution to barrier dysfunction. *In vivo,* wild type and TLR4^−/−^ transgenic mice were used to study the role of TLR activation by SEB in increasing mucosal permeability for FD4.

**Results**: TER of CRSwNP cultures was significantly decreased compared to controls, which was associated with decreased expression of occludin. Stimulation with SEB significantly decreased TER in CRSwNP cultures associated with decreased occludin, and had no effect in control cultures. TLR2 and TLR4 expression was elevated in CRSwNP, which could explain the differential SEB response. Moreover, occludin levels correlated inversely with TLR4 expression in CRSwNP. Antagonizing TLR signaling prevented SEB-induced decrease in TER in vitro*. In vivo,* SEB increased mucosal permeability for FD4 in wild type mice by affecting the expression of occludin. No increased FD4 permeability nor decreased expression of occludin was found in TLR4^−/−^ transgenic mice.

**Conclusions**: TLR4 expression is increased in CRSwNP and correlated inversely with occludin levels. TLR4 seems a key factor in regulating barrier integrity in CRSwNP.

**Keywords**: TLR4; CRSwNP; Epithelial Integrity; Tight Junction

### O07 Investigation of the Th17 inflammatory response in chronic rhinosinusitis

#### Timothy C. Biggs^1^, Stephen M. Hayes^1^, Philip G. Harries^2^, Sylvia Pender^1^, Rami J. Salib^1^

##### ^1^University of Southampton, Southampton, United Kingdom; ^2^University Hospital Southampton NHS Foundation Trust, Southampton, United Kingdom

**Correspondence**: Timothy C. Biggs - t.biggs@soton.ac.uk

*Clinical and Translational Allergy* 2017, **7(Suppl 3)**:O07

**Introduction**: T-helper 17 (Th17) cells represent a distinct T cell linage, involved in the host defence against extracellular pathogens. There have been limited studies of a Th17 mediated inflammatory response in chronic rhinosinusitis patients. We aimed to study Th17 responses in chronic rhinosinusitis with and without nasal polyps (CRSwNP and CRSsNP respectively) including stimulation with *Staphylococcus aureus* enterotoxin B (SEB).

**Methods**: In total, 31 patients were included within the study. Nasal polyp, CRSsNP sinonasal mucosa and control mucosa was harvested at the time of surgery. Th17 cytokine gene expression was assessed using real time quantitative polymerase chain reaction (RT-qPCR). In addition, nasal polyp and control tissues were cultured with SEB for 24 h using an ex vivo nasal explant model, prior to cytokine analysis using RT-qPCR and Luminex.

**Results**: IL-17A gene expression was significantly upregulated in CRSsNP sinonasal mucosa compared to nasal polyps (p < 0.05) and control mucosa (p < 0.01). Upon stimulation with SEB, IL-17A gene expression and protein production was significantly upregulated (p < 0.05) within nasal polyps.

**Conclusions**: This study demonstrates a Th17 response in CRSsNP sinonasal mucosa, and in nasal polyps when stimulated with SEB. These results highlight the likely role of bacteria and their toxins in driving the inflammatory response in CRS, resulting in disease resistance and long term chronicity.

### O08 Regulation of hyperplastic cell growth in chronic rhinosinusitis with nasal polyposis

#### Jean Kim, Hyun Sil Lee

##### Johns Hopkins University School of Medicine, Baltimore, United States

**Correspondence**: Jean Kim - jeankim@jhmi.edu

*Clinical and Translational Allergy* 2017, **7(Suppl 3)**:O08

**Introduction**: We have previously shown that VEGF is overexpressed in the epithelium of hyperplastic nasal polyps in patients with chronic rhinosinusitis with nasal polyposis (CRSwNP). We hypothesized that the HIF family of transcription factors function to regulate this process.

**Methods**: We used human primary nasal epithelial cells grown in submerged culture (PNEC) and whole human polyp tissue in air liquid interface culture to examine to examine HIF and VEGF expression by immunohistochemistry, flow cytometry and realtime PCR analysis. Function was assessed by exposure to known pharmacologic inhibitor digoxin and specific siRNA knockdown of HIF on cell growth and apoptosis by DNA binding assay of PNEC and TUNEL assay of polyp tissue, respectively.

**Results**: We now report that the HIF family of transcription factors, both HIF1-alpha (HIF1α) and HIF2-alpha (HIF2α), which known regulators of expression of VEGF, are highly expressed in PNEC from CRSwNP subjects, as compared to normal control subjects when analyzed by flow cytometry of PNEC and immunohistochemistry of sinonasal surgical tissue. PNEC from CRSwNP patients express high constitutive levels of mRNA of HIF1α and HIF2α. Inhibition of HIF1α by cardiac glycoside digoxin results in 65% inhibition of PNEC growth rates analyzed by DNA binding assay (p < 0.001, n = 5). Further studies show that siRNA knockdown of HIF1α results in inhibition of VEGF mRNA expression by 75% (p < 0.01, n = 3) and subsequent 55% inhibition of PNEC growth rate as measured by DNA binding assay (p < 0.01, n = 3). Exposure of whole human nasal polyps in ex vivo culture to HIF1α inhibitor digoxin or VEGF co-receptor inhibitor anti-neuropilin1 antibody results in 90% (p < 0.01, n = 5) and twofold (p < 0.005, n = 5) increase in apoptosis respectively of PNEC as assessed by TUNEL assay.

**Conclusions**: Collectively, these data indicate that HIF regulates VEGF in promoting hyperplastic nasal epithelial cell growth and survival observed in CRSwNP.

**Keywords**: Nasal Polyposis, Epithelial Cell Growth, VEGF, HIF

### O09 Interleukin 7 and Interleukin 15 interaction with the inflammation severity, predisposing factors and phenotype in chronic rhinosinusitis

#### Livije Kalogjera^1^, Nada Vrkic^2^, Anita Topic^3^, Dejan Tomljenovic^4^, Tomislav Greguric^5^, Patricija Bankovic Radovanovic^6^

##### ^1^ORL/HNS Dept. University Hospital Centre, Zagreb, Croatia; ^2^Biochemistry Lab,. University Hospital Centre, Zagreb, Croatia; ^3^Clinical Institute of Chemistry, Zagreb, Croatia; ^4^ORL/HNS Dept, University Hospital Centre, Zagreb, Croatia; ^5^Clinical Dept of Radiology, University Hospital Centre, Zagreb, Croatia; ^6^Dept of Clinical Chemistry, Genera Hospital Pula, Pula, Croatia

**Correspondence**: Livije Kalogjera - kalogjera@sfzg.hr

*Clinical and Translational Allergy* 2017, **7(Suppl 3)**:O09

**Introduction**: Two major clinical phenotypes of chronic rhinosinusitis (CRS), with (CRSwNP) and without nasal polyps (CRSsNP) may show different regulation of inflammation, interplay between T cell subsets, remodelling, response to microbiome and association with predisposing comorbidities, like allergy, asthma and aspirin intolerance. IL-7 and IL-15 belong to common gamma chain cytokines, and are essentially involved in T cell homeostasis and proliferation. Several papers indicated that they might be involved in the immune response in asthma, however, their role in CRS was not evaluated. Our aim was to analyze interaction between IL-7 and IL-15 related to phenotypes and comorbidities.

**Methods**: The study included 50 patients(both gender, age 18–60 years, 32 with CRSsNP, and 18 with CRSwNP), with CRS according to the EPOS criteria, who were submitted to endoscopic sinus surgery. Ethmoid sinus mucosa or nasal polyps were collected at surgery and immediately tissue homogenates were prepared and incubated with protease inhibitor. Assays (R&D System, Quantikine ELISA, UK) for interleukins IL-4, IL-5, IL-7, IL-15 and for interferon gamma (IFN-γ) employs the quantitative sandwich enzyme immunoassay technique. Tissue homogenates (n = 70) were analysed according to the manufacurer’s instructions (29 from non-allergic CRSsNP, 17 from allergic asthma patients, 11 from non-allergic asthma, 9 from allergic non-asthmatic CRSsNP and 4 from aspirin intolerant CRSwNP). Polyp and ethmoid sinus mucosa samples were taken in CRSwNP, and only ethmoid sinus mucosa from patients with CRSsNP, respectively, from the worse side according to imaging.

**Results**: Results. IL-5 was significantly higher (mean 38.69 vs. 9.47 pg/mL respectively), but IL-7 (0.1397 vs. 0.2638 pg/mL, respectively), and IFN-γ (22.44 vs. 41.71 pg/mL, respectively) significantly lower in samples from CRSwNP, and these differences were also significant when asthmatic were compared to non-asthmatic, irrespective of phenotype and sensitization. Interestingly, IFN-γ was significantly higher in patients with allergic sensitization, while IL-4 was significantly higher in patients with ASA intolerance. IL-15 significantly correlated with IL-7 (rho 0.44), IL-4 (rho 0.31) and IFN-γ significantly with IL-4 (rho 0.51).

**Conclusions**: IL-7 and IL-15 are significantly upregulated in CRSsNP compared to CRSwNP and in non-asthmatics compared to asthmatics.

**Keywords**: IL-7, IL-15, Chronic Rhinosinusitis, Nasal Polyps, T Cell Subset

### O10 Problems and solutions in the treatment of acute and chronic rhinosinusitis

#### Claus Bachert, Rainer Jund

##### URL, Ghent, Botswana

**Correspondence**: Claus Bachert - claus.bachert@ugent.be

*Clinical and Translational Allergy* 2017, **7(Suppl 3)**:O10

**Introduction**: Acute and chronic rhinosinusitis affect everyone several times per year and 11% of the EU population, respectively.

**Methods**: Double-blind randomized placebo-controlled trials.

**Results**: Recently, the herbal drug BNO 1016 showed evidence for a significant and clinically relevant reduction of symptoms and increase of quality of life. In an analysis of pooled data of two similar randomized placebo-controlled clinical trials including 589 patients, the Major Symptom Score (MSS) improved from 10.02 ± 1.61 to 2.47 ± 2.55 for BNO 1016 and from 9.87 ± 1.52 to 3.63 ± 3.63 for placebo; the difference between treatment groups at end of therapy (1.16 score point ± [SD]) was statistically significant in favor of BNO 1016 (p < 0.0001); patient-assessed quality of life also improved significantly (p = 0.0015) versus placebo. The results were confirmed by ultrasonography. In summary, the herbal dry extract BNO 1016 is efficacious and well tolerated in patients with acute viral rhinosinusitis.

The objective of a recent clinical trial therefore was to assess the efficacy, safety and tolerability of dry extract BNO 1016 in patients with chronic rhinosinusitis. 927 patients suffering from chronic rhinosinusitis (>12 weeks and 6 ≤ MSS ≤ 12) were enrolled in a randomised placebo-controlled trial with a treatment period of 12 weeks. The primary endpoint was the mean Major Symptom Score (MSS) over week 8 and week 12 compared to placebo. Secondary endpoint included further MSS related calculations and responder rates over time. Finally, safety and tolerability were evaluated. Although the results of the secondary endpoints showed a clear trend towards superior efficacy, BNO 1016 was not superior over placebo regarding the primary endpoint. Additional post hoc sensitivity analyses in patients with a baseline MSS ≥ 10, duration of disease > 1 year and diagnosed by specialists in otorhinolaryngology indicated that those patients significantly benefited from BNO 1016. Therapy was superior for the primary endpoint analysis. Patients were less impaired with respect to work and daily activities. A good safety and tolerability of BNO 1016 was assured in all patients.

**Conclusions**: Rhinosinusitis, a frequent cause of disease and associated sequalae, still remains a challenge for treatment. BNO 1016 can safely be administered in patients with acute and chronic rhinosinusitis and offers a suitable treatment option.

**Keywords**: Acute Rhinosinusitis, Chronic Rhinosinusitis, Phytotherapy, BN1016

## Oral Abstract Session 6: CRS & Aspirin exacerbated respiratory disease (AERD)

### O11 Transglutaminase 2 expression but not lipid mediator profiles discriminate nasal polyps from steroid treated aspirin tolerant and intolerant patients

#### Pascal Haimerl^1^, Adam M. Chaker^2^, Yvonne Schober^3^, Sonja Schindela^1^, Andreas Nockher^3^, Carsten B. Schmidt-Weber^1^, Julia Esser-Von Bieren^1^

##### ^1^Center of Allergy and Environment (ZAUM), Technical University of Munich and Helmholtz Center Munich, Munich, Germany; ^2^Department of Otolaryngology, Allergy Section, Klinikum Rechts der Isar; Center of Allergy and Environment (ZAUM), Technical University of Munich, Munich, Germany; ^3^Institute of Laboratory Medicine and Pathobiochemistry, Molecular Diagnostics, Philipps University Marburg, Marburg, Germany

**Correspondence**: Julia Esser-Von Bieren - julia.esser@tum.de

*Clinical and Translational Allergy* 2017, **7(Suppl 3)**:O11

**Introduction**: Aspirin exacerbated respiratory disease (AERD) is characterized by acute aspirin-triggered respiratory distress, chronic asthma and nasal polyps that are often refractory to steroid treatment. AERD patients show characteristic abnormalities in lipid mediator (eicosanoid) metabolism and signaling, but the mechanisms that discriminate nasal polyp development in AERD and aspirin tolerant chronic rhinosinusitis with nasal polyps (AT CRSwNP) remain obscure. We thus compared eicosanoid profiles in nasal polyps from AERD and AT CRSwNP patients and analyzed the expression of factors involved in airway remodeling.

**Methods**: Nasal polyp tissues (n = 10 AT CRSwNP, n = 4 AERD) were obtained from patients undergoing functional sinus surgery. All patients were treated with systemic steroids preoperatively. Expression of eicosanoid pathway proteins and regulatory factors in nasal polyp tissues or healthy turbinates (n = 3) was studied by immunofluorescence and immunohistochemistry followed by automated image analysis. Supernatants from overnight cultures of nasal polyps were processed and subjected to lipid mediator analysis by LC–MS/MS or immunoassay.

**Results**: Nasal polyp tissues from steroid treated AT CRSwNP and AERD patients expressed high levels of leukotriene (LT) biosynthetic enzymes (5-lipoxygenase, Leukotriene C4 synthase) despite low levels of infiltrating granulocytes. LT enzymes were abundant in the nasal polyp epithelium and in macrophages and all nasal polyp tissues released significant amounts of LTs. The overall eicosanoid profile was similar in nasal polyps from AT CRSwNP and AERD patients with a tendency for increased thromboxane production by AERD tissues. In contrast AERD tissues expressed significantly lower levels of the enzymes transglutaminase 2 (TG2) and microsomal prostaglandin E2 synthase (mPGES-1). TG2 was particularly abundant in tissues from house dust mite allergic patients.

**Conclusions**: Our findings identify TG2 as a potential mechanism that discriminates nasal polyp endotypes. We further show that TG2 expression and LT production in nasal polyp epithelium are resistant to steroid treatment. Thus, targeting TG2 might be promising in allergic CRSwNP patients and novel strategies targeting steroid resistant lipid mediator abnormalities in nasal polyps are urgently needed.

**Keywords**: AERD, Allergy, Eicosanoids, Nasal Polyps, Transglutaminase 2

## Poster Discussion Session 2: CRS and Omics

### P12 Effects of Cytokine secretion from nasal polyps on peripheral lymphocytes in a co-culture system

#### Pascal Ickrath^1^, Norbert Kleinsasser^1^, Niklas Beyersdorf^2^, Xin Ding^2^, Rudolf Hagen^1^, Stephan Hackenberg^1^

##### ^1^Department of Otorhinorhinology, University of Wuerzburg, Wuerzburg, Germany; ^2^Institute of Immunology and Virology, Wuerzburg, Germany

**Correspondence**: Pascal Ickrath - ickrath_p@ukw.de

*Clinical and Translational Allergy* 2017, **7(Suppl 3)**:P12

**Introduction**: T cell subpopulations in nasal polyps differ from peripheral lymphocytes in patients with CRSwNP. The direct influence of the nasal polypoid tissue by secretion of cytokines on the differentiation or activation of T cells still remains unclear. To study the effects of polypoid tissue on peripheral lymphocytes in vitro, we developed a co-culture system with nasal polyp tissue and peripheral lymphocytes to directly measure these interactions. Additionally, we assessed the cytokine secretion by polyp tissue, which may induce T cell responses in this system.

**Methods**: Tissue and blood samples were collected from 10 patients undergoing nasal sinus surgery. Polypoid tissue was cultured under air–liquid interface conditions. Afterwards, CD3/CD28 activated PBMC of the same patients were added using cytokine free medium. After 3 days, lymphocytes were separated and analyzed by multicolor flow cytometry. Monoculture PBMC served as control group. Additionally, cytokine secretion of the polyp tissue was measured using a human Th1/Th2/Th17 antibody array.

**Results**: There was a significantly higher amount of CD4^+^ and CD8 + T cells in the co-cultured system than in PBMC alone. Terminal differentiated CD8 + T cells were significantly increased, while central memory CD8 + T cells significantly decreased in the co-culture. HLA-DR was downregulated in co-cultured CD3 + lymphocytes. Conventional memory CD4 + T cells significantly increased and resting regulatory T cells significantly decreased in the co-cultured system. Cytokine analysis showed a secretion of IL-6, GM-CSF and MIP-3.

**Conclusions**: It could be demonstrated that nasal polypoid tissue has an effect on the PBMC phenotype. The secretion of pro-inflammatory cytokines may play a regulatory role in this context. Interestingly, the clinically known downregulation of HLA-DR in CD3 + lymphocytes was significantly reproducible in vitro. The modulating effect of the polypoid tissue on the activation of lymphocytes must be further investigated in order to determinate its impact on this disease.

**Keywords**: Chronic Rhinosinusitis With Nasal Polyps, T Cell Subsets, Co-Culture System

### P13 A new cytological approach to improve the final diagnosis of Eosinophilic Granulomatosis with Polyangiitis (EGPA)

#### Daniela Cangiano^1^, Francesco Cinetto^1^, Giuseppe Brescia^2^, Gino Marioni^2^, Claudia Zanotti^2^, Franco Schiavon^3^, Roberto Padoan^3^, Ilaria Caputo^4^, Raffaella Neri^1^, Carlo Agostini^1^

##### ^1^Department of Medicine DIMED,University of Padua, Padova, Italy; ^2^Department of Neurosciences DNS, Otolaryngology Section,University of Padua, Padova, Italy; ^3^Department of Internal Medicine, University of Padua, Padova, Italy; ^4^University of Padua, Padova, Italy

**Correspondence**: Daniela Cangiano - cangiano.daniela@gmail.com

*Clinical and Translational Allergy* 2017, **7(Suppl 3)**:P13

**Introduction**: Eosinophilic granulomatosis with polyangiitis (EGPA) is an uncommon systemic necrotizing vasculitis that affects small to medium sized vessels and is associated with severe asthma, allergic rhinitis, nasal polyposis and blood and tissue eosinophilic infiltration. Antineutrophil cytoplasmic autoantibodies (ANCA) are present in about 40% of cases. The presence of four or more of the above described findings yields a sensitivity of 85% and a specificity of 99.7% for the final diagnosis of EGPA. The aim of the study was to develop a new diagnostic tool to support the diagnostic and prognostic work-up of EGPA patients with the possibility to evaluate extracellular mediators in nasal secretion.

**Methods**: Nasal secretions were gained from 40 patients of which 20 EGPA, 10 suspected EGPA and 10 controls after positioning the cotton pieces in the nasal middle meatus where they were left for 10 min. Subsequently the cotton pieces were put in 5 ml of saline solution and left in ice until processing. The liquid obtained from the squeezing of the cottons was centrifuged two times at 1600 RPM for 10 min. Supernatant was frozen after first centrifugation. Trypan Blue was added to 10 μl of sample to calculate the number of cells and their vitality. Finally, 100 μl of sample with containing 1.5–2.00 × 10^5^ cells were centrifuged with Cytospin at 300 RPM for 15 min. The slides were stained with May Grunwald/Giemsa and cell population analysed at microscope with objective oil immersion 40× of magnification. The remaining cells were frozen or cultured.

**Results**: The mean number of cells obtained after centrifugation was 2.00–4.08 × 10^6^ cells/ml for EGPA patients and 6.42–8.62 × 10^5^ cells/ml for control group and suspected EGPA. Cytologic analysis allowed us to count an increase in the percentage of eosinophils in EGPA patients with active ENT disease and in some of the patients with a suspect of EGPA, where this percentage correlated with histologic evidence of disease.

**Conclusions**: This method seems preliminarily a reliable cytologic examination allowing fresh cells isolation and analysis of extracellular mediators. Compared to the direct slither of the mucus on the surface of the glass, cytospin significantly reduced any dye accumulation. Further studies are ongoing to support the described method, in order to confirm the cytologic diagnostic reliability to identify prognostic biomarkers of EGPA.

### P14 Enhanced Type I Interferon response contributes to Eosinophilic chronic rhinosinusitis

#### Ji Heui Kim, Yong Ju Jang, Ji Youn Lim, Sung Hee Kim

##### Asan Medical Center, Seoul, South Korea

**Correspondence**: Sung Hee Kim - tomato831@daum.net

*Clinical and Translational Allergy* 2017, **7(Suppl 3)**:P14

**Introduction**: Type I interferons (IFN-I) response has been implicated in the eosinophilic inflammation besides its antiviral function. This study aimed to investigate the role of IFN-I response in the pathogenesis of eosinophilic chronic rhinosinusitis (ECRS).

**Methods**: The expressions of IFN-I and IFN-I receptor (IFNAR1) in sinonasal tissue from controls and patients with CRS with nasal polyp (NP) were measured using real time-PCR, ELISA, and/or immunohistochemistry. The levels of CCL11/eotaxin-1, IL-5, and IL-13 in sinonasal tissue of human subjects were determined by ELISA. ECRS in the wild type (WT) and IFNAR1 knockout (*Ifnar1* ^−/−^) mice was induced by intranasal challenge of aspergillus protease plus ovalbumin (OVA). Stromal cells cultured from NP tissue were stimulated by exogenous IFN-β and their CCL11/eotaxin-1 production was measured by ELISA.

**Results**: IFN-β, IFNAR1, IL-5, IL-13, and CCL11 expressions were increased in NP tissues from ECRS compared with uncinated process mucosa from controls. IFN-β levels positively correlated with IL-13 and CCL11 levels. The histological severity of *Aspergillus* protease plus OVA induced-ECRS was less in *Ifnar1* ^−/−^ than in WT mice. The levels of IL-4, IL-5, and CCL11 in the nasal lavage fluid of *Ifnar1* ^−/−^ mice were significantly lower than those in WT mice. The serum total IgE levels in *Ifnar1* ^−/−^ mice were also lower than those in WT mice. NP stromal cells produced significantly greater amount of CCL11/eotaxin-1 by concentration-dependent stimulation of IFN-β.

**Conclusions**: Our results showed that IFN-I response was up-regulated in the NP tissues from ECRS, IFN-β increased CCL11/eotaxin production in the NP stromal cell culture model, and development of ECRS was inhibited by deficiency in IFN-I signaling. Therefore, increased IFN-I response in the sinonasal mucosa may underlie in the pathogenesis of ECRS.

**Keywords**: Chronic Rhinosinusitis, Type I Interferon, Eosinophil

### P15 In search of diverse endotypes of chronic rhinosinusitis with Nasal polyps in patients living in different geographical regions of the Russian Federation: pilot study

#### Elena Savlevich^1^, Leonid Gaganov^2^, Maria Kochnova^2^, Victor Egorov^2^

##### ^1^Central State Medical Academy of Department for Presidential Affairs of the Russian Federation, Moscow, Russia; ^2^Moscow Regional Research and Clinical Institute (MONIKI), Moscow, Russia

**Correspondence**: Elena Savlevich - savllena@gmail.com

*Clinical and Translational Allergy* 2017, **7(Suppl 3)**:P15

**Introduction**: Chronic rhinosinusitis with nasal polyps (CRSwNP) affects 4% of the total population (C.A. Akdis, 2013). The prevalence of CRSwNP in Russian Federation (RF) is 1.5% or 4.9 per 10,000 population (Lopatin A, 2014). The ratio and number of neutrophils and eosinophils in the vast majority of nasal polyps (NP) are diverse. According to the data from research neutrophilic types of adult bilateral polyps do occur, predominantly in Asian subjects and some populations in North America (Zhang N, 2006). Russia is a multiethnic country that separates Europe and Asia. It is of interest as a target for epidemiological studies.

The aim was to explore the histology of NP biopsies obtained from operated patients with CRSwNP living in different regions of RF.

**Methods**: NP samples obtained from 152 patients during polypectomy were assaying histologically to identify neutrophils and eosinophils count and ratio of eosinophils to neutrophils (ENR) in biopsies. There were 10–11 samples of polyp’s tissue from 15 ENT hospitals from different regions of the country. There was equal quantity of participants of 12 nationalites belonging to Caucasian or Mongoloid race. All patients completed a questionnaire including data about symptoms, nationality, comorbidities, atopic reactions and drug hypersensitivity.

**Results**: There was predominance of eosinophil’s infiltration (>90% of all cells) in all NP samples. There were no significance between clinical symptoms and NER. The NP with predominant eosinophil’s count (ENR median total = 6.8) were isolated from Siberian and Volzhsky region habitants (Tomsk ENR = 23.3, Irkutsk = 9.14, Nizhniy Novgorod = 11.3). There were no significant differences in ENR in patients of different nationalities. Moreover the predominance of eosinophilic infiltration in Russian (Caucasoid) were revealed in 69.5% of cases versus patients with other nationalities patients (83%). In most biopses from Russian patients ENR = 6.0 in comparison to others ENR = 6.9.

**Conclusions**: There’re some hypotheses about triggering factors of CRSwNP. The most popular one is the influence of microbial agents (C. Bachert). Probably the features of infectious and parasitic diseases (zoonoses, insect bites, ticks, infected fish, etc.) and climate factors are cause of debut of formation of polyps. Russia is excellent model for epidemiological studies that could reveal these factors.

**Keywords**: Nasal Polyp, CRSwNP, Eosinophils, Endotypes

### P16 Eosinophilic oesophagitis with sinonasal polyposis: a single eosinophilic disorder?

#### Jie Shen Fok

##### Flinders Medical Centre, Bedford Park, Australia

**Correspondence**: Jie Shen Fok - fulfilled1978@yahoo.com

*Clinical and Translational Allergy* 2017, **7(Suppl 3)**:P16

**Introduction**: Is concurrent eosinophilic oesophagitis and sinonasal polyposis a pure coincidence? Or, do they share common pathogenesis? We report a case series of four subjects with both conditions managed at a local allergy clinic in Adelaide, South Australia, Australia, highlighting certain features common to both.

**Methods**: N/A.

**Results**: All four subjects ranged from age 50 to 61 at the time of presentation, seeking treatment for chronic nasal symptoms, which were managed predominantly with intranasal corticosteroids. All four were asthmatic and have had intranasal surgery in the past, with two of them having had four surgeries to date. Results of polyp biopsy were not available. Peripheral eosinophilia was characteristic of all four, ranging from 0.6 to 1.56 × 10^9^. Background of atopy was further evaluated with skin prick testing with aeroallergens and food allergens with no concordant findings noted across all four subjects. Nasal symptoms preceded oesophageal symptoms by 2 to 3 years. All subjects had oesophageal biopsy confirming eosinophilic oesophagitis. Three subjects were aspirin sensitive. Aspirin or sodium salicylate desensitisation was tried without success. Two subjects responded to low salicylate diet with the remaining two not yet trialled on such. Three subjects responded symptomatically to montelukast.

**Conclusions**: Is there a single factor driving the pathomechanistic pathway e.g. IL-5? Nasal polyposis occurs frequently in patients with intolerance to salicylate acid and those with allergic fungal sinusitis. It has been demonstrated that IL-5 has a key role in the pathophysiology of eosinophil dominated polyps. Additionally, adhesion molecules VCAM-1 is recognised to play a role in the extravasation of eosinophils into nasal polyps and cytokines such as IL-3, IL-4 and TNF-a can induce VCAM-1 expression in microvascular endothelium from the polyps. Future planned testing includes eosinophil activation markers on flow cytometry, along with serum levels of cytokines mentioned.

Further questions then arise for the management of future patients, especially in regards to the eosinophilic oesophagitis component. Is it worthwhile to have six food elimination diet based on skin prick testing results, followed by a low salicylate diet? In the case where aspirin sensitivity is confirmed, is it worthwhile to have a direct move to low salicylate diet? Is it worthwhile to attempt sodium salicylate desensitisation on all aspirin sensitive or dietary salicylate sensitive patients?

**Keywords**: Eosinophilic Oesophagitis, Sinonasal Polyposis, Eosinophilic Disorder

### P17 Extended draf IIb.2 Procedure in the treatment of unilateral frontal sinusitis

#### Tengchin Wang

##### Department of Otolaryngology, Tainan Municipal Hospital, Tainan City, Taiwan

**Correspondence**: Tengchin Wang - tengchin27@hotmail.com

*Clinical and Translational Allergy* 2017, **7(Suppl 3)**:P17

**Introduction**: Traditionally, Draf III procedure is considered for accessing the refractory pathogens inside the frontal sinus. In case of confined, predominantly unilateral lesions, Draf IIb procedure also provides wide access to the frontal sinus. This approach can be extended without destruction of the contralateral frontal sinus drainage pathway.

**Methods**: We report on a patient suffering from left frontal pain and nasal obstruction for 6 months. He received examination in our department, and fiberscopy revealed polyposis. Computed tomography displayed the over-pneumatized right frontal sinus pushing intersinus septum toward to left side and causing narrowed left frontal space. We decided to performe Draf IIb.2 procedure.

**Results**: Once the frontal recess is identified, we started using the irrigated RAD curved burr (3.6 mm 55°) to remove partial anterior–superior portion of middle turbinate and frontal beak. After identifying intersinus septum, we removed its lower part and formed a drainage pathway through rightl frontal sinus. There were no sequelas and patient felt well during follow-up.

**Conclusions**: In case of confined, unilateral frontal lesions, less destructive, limited approaches, defined as extended Draf IIb, can be applied without disturbing the contralateral frontal sinus drainage pathway according to the literatures. Gotlib et al. have classified extended Draf IIb procedure into 3 subtypes; Extended Draf IIb.2 (or mini-Lothrop) refers to Draf IIb procedure with removal of the lower intersinus septum. This technique is applicable for bilateral frontal lesions with one side limiting in frontal sinus or unilateral frontal sinus disease with intersinus septum deviation towards the lesion. It can minimiz destruction of the contralateral frontal sinus drainage pathway when comparing to Draf III. The nasal septum can be kept intact as compared to Draf IIb.1 and Draf IIb.3 procedures.

**Keywords**: Draf IIb Procedure

### P18 Nasal epithelial transcriptome in subjects with birch pollen allergic rhinitis: evaluation of the seasonal and immunotherapy-related alterations

#### Tanzeela Hanif, Jutta Renkonen, Sakari Joenväärä, Matti Kankainen, Mika Mäkelä, Paula Kauppi, Anna Pelkonen, Pirkko Mattila, Risto Renkonen, Sanna Toppila-Salmi

##### University of Helsinki, Helsinki, Finland

**Correspondence**: Tanzeela Hanif - tanzeela.hanif@helsinki.fi

*Clinical and Translational Allergy* 2017, **7(Suppl 3)**:P18

**Introduction**: Birch pollen allergic rhinitis (AR) is one of the common allergic diseases in Europe. Little knowledge exists on changes in nasal epithelial transcriptome during allergen exposure or allergen specific immunotherapy. The aim of this study was to evaluate the seasonal and immunotherapy-related alterations in nasal epithelial transcriptome in subjects with or without birch pollen allergic rhinitis (AR).

**Methods**: The study subjects were healthy nonsmoking adults with or without birch pollen AR (N = 11). We used the RNA sequencing to demonstrate the alterations in transcriptome of study subjects. The whole study spanned on two years i.e. 4 seasons, where half of the AR patients started the immunotherapy during the second year. We used edgeR for differential expression analysis and R environment for further Bioinformatics. Gene Ontology (GO) and KEGG pathway enrichment was done using DAVID (Database for Annotation, Visualization, and Integrated Discovery).

**Results**: We found a significant change in gene expression of the nasal epithelial cells in AR patients not having the immunotherapy compared to healthy controls. We found immunity related GO terms up or down -regulated in these patients. We also observed variations in biological processes of IL-2 production, cytokines production, regulation of lymphocyte and leukocyte differentiation. Immunotherapy induced a down regulation of GO terms related to immune response in AR patients.

**Conclusions**: Birch pollen AR induces in-seasonal changes in gene expression. It can alter the regulation of immunity related functions and pathways in AR patients. Immunotherapy might normalize the barrier function by decreasing epithelial immune response. More research with larger number of samples from different populations is needed.

**Keywords**: Allergic Rhinitis, Birch Pollen, Gene Ontology, Immunotherapy, KEGG

## Oral Abstract Session 7: Allergen Immunotherapy - Hot Topics

### O12 Subcutaneous house dust mite immunotherapy is associated with a long-term suppression of allergen-induced basophil activation: a prospective study

#### Margot Berings^1^, Natalie De Ruyck^1^, Lara Derycke^1^, Gabriele Holtappels^1^, Claus Bachert^1^, Bart N. Lambrecht^2^, Melissa Dullaers^2^, Philippe Gevaert^1^

##### ^1^Upper Airways Research Laboratory, Ghent University, Ghent, Belgium; ^2^Laboratory of Immunoregulation, VIB Inflammation Research Center, Ghent University, Ghent, Belgium

**Correspondence**: Margot Berings - margot.berings@ugent.be

*Clinical and Translational Allergy* 2017, **7(Suppl 3)**:O12

**Introduction**: The effect of allergen-specific immunotherapy (IT) on basophils remains incompletely understood. Several studies have shown an early suppression of basophil activation, and a few studies have reported long-term suppression in response to IT. These studies involved venom, grass, birch or cat IT. To our knowledge, no previous studies investigated the effect on basophil activation in the context of house dust mite (HDM) IT. Objectives: To study the effect of HDM IT on allergen-stimulated basophil activation and to evaluate its relation with clinical response to treatment.

**Methods**: Patients with HDM allergic rhinitis undergoing conventional HDM subcutaneous IT (HDM SCIT, n = 23) and patients not undergoing IT (HDMA, n = 10) were studied prospectively. Basophil activation experiments were performed before start of IT (*visit 0*), halfway initiation (*visit 1*), at the end of initiation (*visit 2*), after 4 months of maintenance (*visit 3*), and after one year of treatment (*visit 4*). The same time line was followed for the HDMA subjects. Whole blood samples were stimulated with various concentrations (0.0023–22.7 ng/ml) of Dermatophagoides pteronyssinus extract. Activated basophils were identified with flowcytometry by expression of CD63. Dose–response curve metrics (EC50, CD-sens, and Area Under the Curve) were determined as measures for basophil allergen-sensitivity.

**Results**: HDM IT induced a significant decrease of basophil activation over time (p < 0.001; change observed from *visit 2*, most marked at *visit 3,* maintained at *visit 4*). In the patients not undergoing IT no significant changes in basophil activation were observed (p = 0.523) and the difference between both groups was significant (HDM SCIT vs. HDMA, p = 0.005). After one year of treatment with IT, three patients reported “no change”, five patients “a little improvement”, six patients “improvement”, and nine patients “large improvement” of allergy symptoms. None of the patients reported worsening of symptoms. Within the HDM SCIT group, the change in allergen-stimulated basophil activation was not correlated with the change in patient-reported symptom control (VAS score, r_s_ = 0.26, p = 0.24).

**Conclusions**: A long-term decrease of allergen-stimulated basophil-activation was observed in response to treatment with house dust mite subcutaneous immunotherapy. The degree of change in basophil activation was however not associated with the degree of symptom improvement.

**Keywords**: Allergy, Rhinitis, Immunotherapy, House Dust Mite, Basophils

### O13 Specific subcutaneous immunotherapy with a depigmented polymerized phleum pratense extract in local allergic rhinitis patients, a double-blind placebo-controlled trial

#### Carmen Rondón^1^, Natalia Blanca-López^2^, Ibon Eguiluz^1^, Paloma Campo^1^, Maria Carmen Plaza^3^, Miguel Gonzalez-Visiedo^3^, Raquel Jurado^3^, Cristobalina Mayorga^3^, Maria Jose Torres^1^, Gabriela Canto^2^, Miguel Blanca^1^

##### ^1^Allergy Unit. Regional University Hospital of Malaga, UMA, Málaga, Spain; ^2^Allergy Service. Hospital Infanta Leonor, Madrid, Spain; ^3^Research Laboratory, IBIMA, Regional University Hospital of Malaga, UMA, Málaga, Spain

**Correspondence**: Carmen Rondón - carmenrs61@gmail.com

*Clinical and Translational Allergy* 2017, **7(Suppl 3)**:O13

**Introduction**: Allergen immunotherapy (AIT) with house dust mite extracts has demonstrated to be an effective treatment in patients with local allergic rhinitis (LAR).

The objective of this study was to evaluate the efficacy of subcutaneous AIT with *Phleum pratense* in LAR patients.

**Methods**: A randomized double-blind, placebo-controlled, parallel-group (DBPCPG), phase II investigator-initiated trial was conducted in 56 patients with LAR to *Phleum pratense*. All subjects provided informed consent, and the Ethic Committee and Spanish Drug Agency approved the study. **clinicaltrials.gov identifier**: NCT02126111.

During the 1st year subjects were randomized to receive subcutaneous AIT with depigmented polymerized 100% *Phleum pratense* allergen extract (LETI, S.L.U. Tres Cantos, Madrid) or placebo. During the 2nd year all received AIT. Differences regarding clinical response (combined symptoms-medication score (CSMS), medication free days), Rhinoconjunctivitis Quality of Life Questionnaire (RQLQ), titrated nasal allergen provocation test (NAPT), skin testing, serum levels of specific-IgG4 and -IgE, and safety between groups were analyzed. Patients were classified as responders (increased tolerance to the allergen in the NAPT) and non-responders (no increased tolerance).

**Results**: During the 1st year AIT induced significant improvements compared to placebo in CSMS (1.2 ± 0.8 vs 2.6 ± 1.2, P = 0.001), number of medication free days (18.1 ± 9.8 vs 4.1 ± 3.3, P < 0.001), RQLQ score (1.98 ± 0.91 vs 3.67 ± 1.27, P = 0.001), and allergen tolerance in NAPT (0.024 ± 0.038 mcg/mL vs 0.003 ± 0.004 mcg/mL, P = 0.010) compared to placebo. The rate of responders was 50%, with negativization of NAPT in 19% of patients treated with AIT. During the 2nd year, a comparable improvement in CSMS, RQLQ and nasal allergen tolerance was observed in both groups. Immunotherapy was well-tolerated, with only six local reactions resolved without treatment.

**Conclusions**: Subcutaneous immunotherapy with this depigmented polymerized allergen extract is a safe and clinically effective treatment for LAR to *Phleum pratense* with clear improvement in disease-specific quality of life.

**Funding**: Institute of Health “Carlos III” of the Ministry of Economy and Competitiveness RETICS ARADyAL (RD16/0006/0001) and Adalusian Health Service SAS 111225.

